# Adult intestinal malrotation: A rare incidental finding in trauma imaging

**DOI:** 10.1016/j.radcr.2026.09.065

**Published:** 2026-10-14

**Authors:** Soheil Mirzaei, Sara Eghbalian

**Affiliations:** a Department of Anatomical Sciences, Faculty of Medical Sciences, Tarbiat Modares University, Tehran, Iran; b Department of Radiology, Shahid Sattari Hospital, Shahid Beheshti University of Medical Sciences, Tehran, Iran; c Department of Radiology, Masih-E Daneshvari Hospital, Shahid Beheshti University of Medical Sciences, Tehran, Iran

**Keywords:** Adult intestinal malrotation, Midgut malrotation, Incidental diagnosis, Computed tomography, Superior mesenteric artery

## Abstract

Intestinal malrotation is a congenital anomaly resulting from abnormal rotation and fixation of the midgut during embryonic development. It is typically diagnosed in infancy, and its detection in adults—particularly in asymptomatic individuals—is rare. In adults, clinical presentation ranges from chronic, nonspecific symptoms to acute intestinal obstruction and midgut volvulus.

A 36-year-old woman presented to the emergency department following a motor vehicle collision. She reported no gastrointestinal complaints and denied any history of recurrent abdominal pain, bilious vomiting, abdominal distension, food intolerance, or symptoms suggestive of bowel obstruction. Contrast-enhanced abdominopelvic CT performed to evaluate traumatic injuries showed no evidence of acute intra-abdominal injury. However, it demonstrated predominantly right-sided small-bowel loops, a left-sided cecum and appendix, an abnormal course of the duodenum, and an anterior position of the superior mesenteric vein relative to the superior mesenteric artery. These findings were consistent with midgut malrotation. There was no evidence of volvulus, obstruction, bowel ischemia, or pneumoperitoneum. The patient was referred to the surgical service for further evaluation and management planning, and she was educated regarding warning signs.

Although midgut malrotation is uncommon in adults, it may be identified incidentally on imaging performed for unrelated indications. Familiarity with the anatomic and mesenteric vascular patterns of this anomaly on CT is essential for accurate diagnosis, prevention of misinterpretation, and timely recognition of potentially life-threatening complications. Management of asymptomatic cases should be individualized based on clinical status, imaging findings, and surgical assessment.

## Introduction

Intestinal malrotation is a congenital developmental anomaly of the midgut that results from partial or complete failure of the normal 270-degree counterclockwise rotation of the bowel around the superior mesenteric artery (SMA), along with inadequate fixation within the peritoneal cavity during embryogenesis [[1], [2], [3], [4], [5], [6]]. This abnormality typically affects both the small and large bowel and may lead to narrowing at the mesenteric root attachment [1,2]. Epidemiologically, the incidence of intestinal malrotation is estimated at approximately 1 in 200–500 live births. However, it becomes symptomatic in only about 1 in 6000 live births [[1], [2], [3],^6^]. Malrotation is classically considered a pediatric disorder, and in 75%–85% of cases it is diagnosed within the first 12 months of life due to acute manifestations such as bilious vomiting, bowel obstruction, or midgut volvulus [4].

In contrast, malrotation in adults is very rare, with a reported prevalence ranging from 0.0001% to 0.19% [4,5]. In adults, this condition often remains asymptomatic and is discovered incidentally during autopsy or unrelated surgical procedures [5,6]. It may also present with nonspecific symptoms such as chronic abdominal pain, bloating, nausea, vomiting, food intolerance, and weight loss [1,4,7]. Because these manifestations are vague, the diagnostic process is often challenging [4,6]. This may lead to delayed diagnosis and multiple referrals across different clinical specialties [8]. Due to its rarity, adult midgut malrotation is frequently diagnosed late, which can markedly increase complications [9,10].

## Case presentation

A 36-year-old woman presented to the emergency department after a motor vehicle collision. After admission, she underwent evaluation according to the standard trauma protocol, including physical examination and additional diagnostic workup. At presentation, she had no specific gastrointestinal complaints. She also denied any history of recurrent abdominal pain, bilious vomiting, abdominal distension, food intolerance, or symptoms suggestive of bowel obstruction.

Abdominopelvic CT was performed to assess for potential traumatic injuries and showed incidental findings of adult midgut malrotation. No evidence of acute intra-abdominal injury was noted (Fig. 1). However, an abnormal arrangement of abdominal viscera and mesenteric vessels was incidentally identified. The superior mesenteric vein (SMV) was located anteriorly to the superior mesenteric artery. This spatial configuration represents a distinct inversion of the normal relative SMA–SMV anatomical relationship, serving as a key diagnostic sign of intestinal malrotation (Fig. 2**A)**. Most small-bowel loops were located on the right side of the abdomen, whereas the cecum and appendix were seen on the left (Fig. 2B). In addition, the duodenum did not follow its expected course and did not demonstrate the usual crossing of the midline. Collectively, these findings favored a diagnosis of adult midgut malrotation. However, due to the non-contrast nature of the CT study, the assessment for bowel wall ischemia was limited, although no obvious signs of ischemia were visible. Bilateral L_5_ spondylolysis was also reported as an associated finding. Given the absence of gastrointestinal symptoms and the lack of imaging evidence of an acute complication, intestinal malrotation was considered an incidental finding during trauma evaluation.

**Fig. 1:**
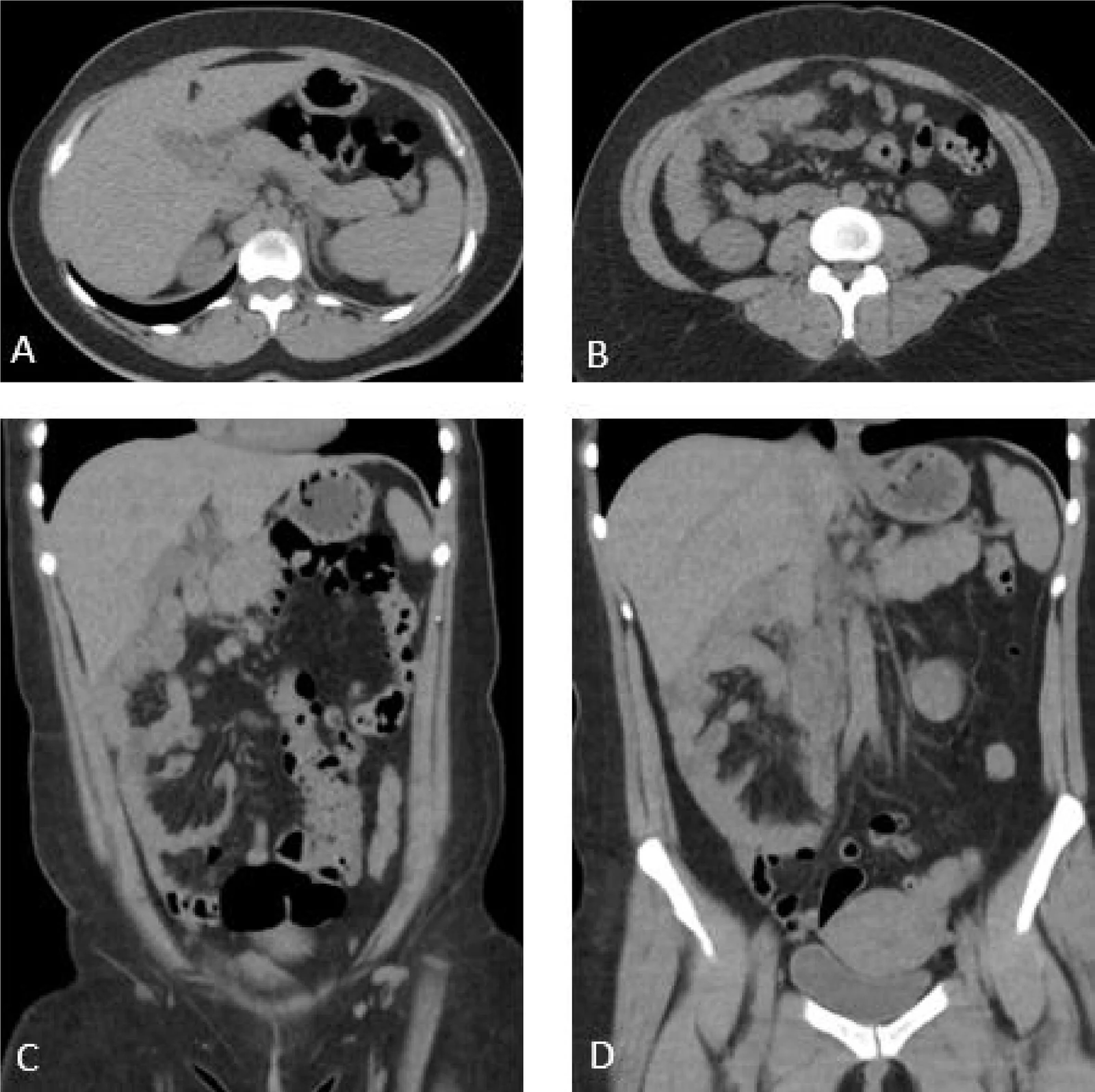
**Abdominopelvic CT findings of incidental adult midgut malrotation**. (A) Axial CT image demonstrating the abnormal arrangement of abdominal viscera, with the majority of small-bowel loops located in the right hemi-abdomen. (**B**) Axial CT image further illustrating the right-sided predominance of the small bowel. (**C and D**) Coronal reformatted CT images confirming midgut malrotation. The abnormal distribution and malposition of bowel loops consistent with midgut malrotation.

**Fig. 2:**
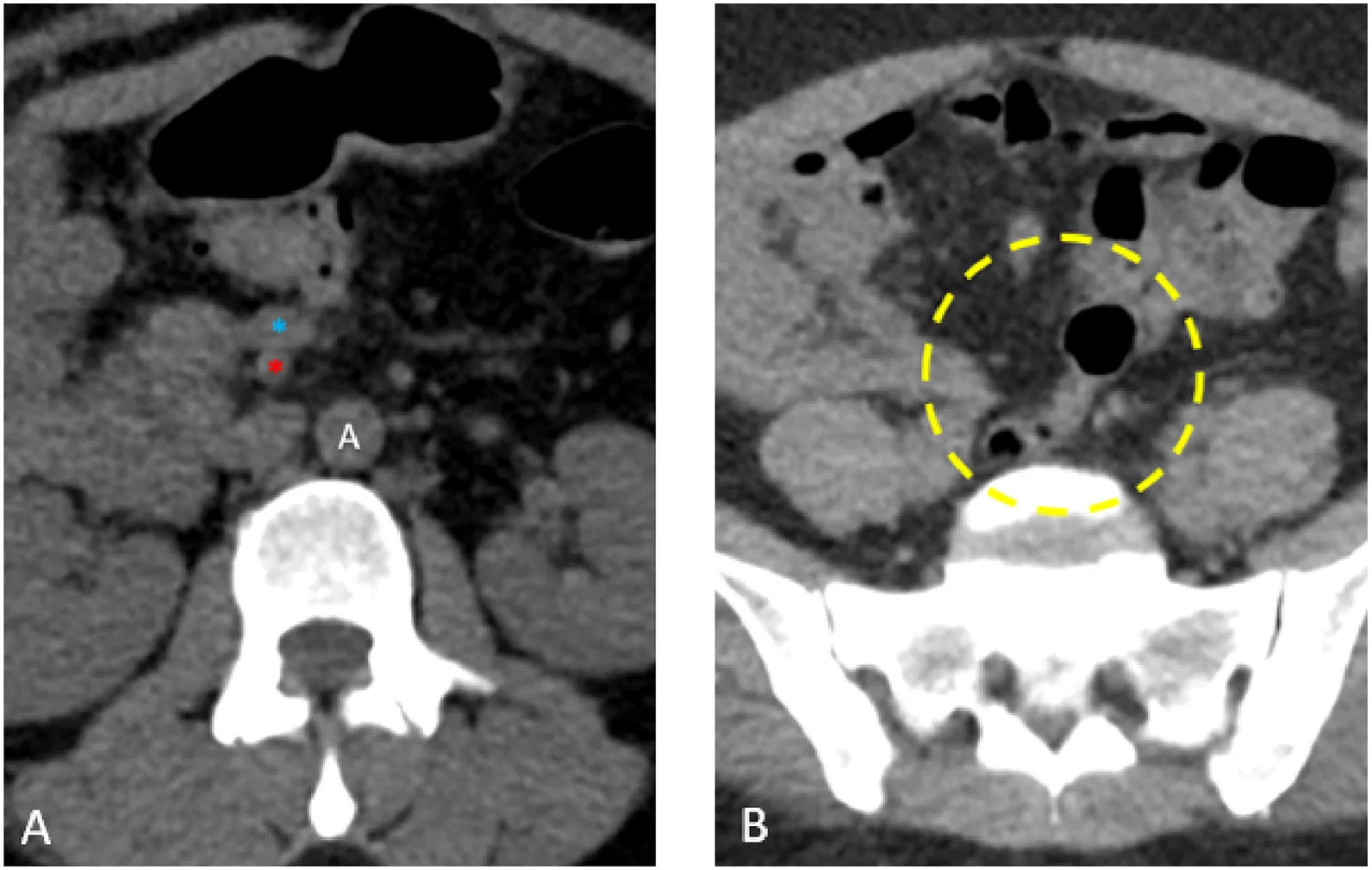
**SMV–SMA inversion in intestinal malrotation with Left-sided cecum and appendix**. ( A) Enlarged axial CT image demonstrating characteristic inversion of the superior mesenteric vascular relationship, with the superior mesenteric vein (SMV; blue asterisk) positioned anterior to the superior mesenteric artery (SMA; red asterisk). A, abdominal aorta. (B) Axial CT image highlighting the cecum and appendix localized to the left side of the abdomen (yellow circle).

After initial assessment and stabilization, the case was discussed with the surgical service to determine appropriate follow-up and possible management. The patient was also counseled regarding the nature of this anomaly and warning symptoms requiring urgent medical attention, including acute abdominal pain, persistent vomiting, abdominal distension, or signs of bowel obstruction.

## Discussion

### Embryology and pathophysiology

The pathophysiologic basis of intestinal malrotation arises from disruption of the embryologic stages of bowel development, which normally occur around the SMA as the primary axis [5,6]. Gut rotation proceeds through three developmental stages. The first stage (weeks 5–10 of gestation) involves physiologic herniation of the midgut into the umbilical cord due to limited intra-abdominal space, accompanied by a 90-degree counterclockwise rotation [1,5].

In the second stage (weeks 10–11), the bowel returns to the abdominal cavity and undergoes an additional 180-degree counterclockwise rotation, completing the total 270-degree rotation [3,5]. The third stage involves descent of the cecum to the right lower quadrant and fixation of the mesentery to the posterior abdominal wall [1,3,5]. Any disruption in these stages may lead to rotational anomalies, including nonrotation—the most common type of malrotation—in which the small bowel is located on the right and the colon on the left side of the abdomen [3,5,6,9].

Disruption of the third stage may also result in a narrow-based, mobile mesentery, rendering the bowel highly susceptible to twisting or volvulus around the SMA [3,7,9]. In addition, abnormal fibrous peritoneal bands known as Ladd’s bands, extending from a malpositioned cecum to the abdominal wall, can mechanically obstruct the duodenum or colon [1,3,4]. Midgut volvulus, by compressing the SMA-associated mesenteric vasculature—particularly venous drainage—can cause congestion, intraluminal hemorrhage, and ultimately bowel gangrene and infarction [5]. Disruption of this sequential rotation leads to an altered mesenteric axis, manifesting radiographically as the anomalous orientation of the SMA andSMV, as well as the abnormal positioning of the duodenojejunal junction.

### Diagnostic approach and imaging findings

Diagnosis of adult intestinal malrotation is difficult because of its rarity and vague clinical manifestations, and it is often delayed by several months [3,4,6,8,10]. Plain abdominal radiography is mainly useful in acute obstruction. On radiographs, absence of cecal gas and clustering of small-bowel loops in the right abdomen may raise suspicion for malrotation [9].

In the pediatric population, upper gastrointestinal contrast study remains the gold standard for diagnosing midgut malrotation, with the pathognomonic finding being the localization of the duodenojejunal junction to the right of the spine [1,4,7,11]. Contrast-enhanced abdominopelvic CT provides substantially higher diagnostic value in adults and is considered the preferred modality [8]. Classic imaging findings include inversion of the SMA/SMV relationship, the whirlpool sign reflecting twisting of bowel and mesentery around the SMA axis, and abnormal bowel positioning [1,2,4,5,[7], [8], [9]]. In normal anatomy, the SMV is positioned to the right of the SMA. An inversion of this relationship—where the SMV lies to the left of, or anterior to, the SMA—is considered a classical computed tomography landmark for intestinal malrotation. Although SMA–SMV inversion is highly suggestive of malrotation, it is not entirely pathognomonic, as normal vessel positioning can occasionally be preserved in incomplete forms, and asymptomatic vascular variations may occur. Nonetheless, when encountered alongside abnormal intestinal disposition (such as a right-sided small bowel and left-sided colon), this vascular inversion provides strong radiological evidence of underlying rotational anomaly, even in incidentally detected cases. The absence of a retro-mesenteric horizontal duodenum is the most accurate CT indicator of midgut malrotation, outperforming traditional imaging signs [12]. Doppler ultrasound may also detect vascular inversion or the whirlpool sign [11]. MR enterography may also assist in diagnosis [8].

### Therapeutic management and surgical challenges

The standard and definitive treatment for intestinal malrotation in both children and adults is the Ladd’s procedure [1,3,10]. This operation includes detorsion of volvulus, division of Ladd’s bands, widening of the mesenteric base, and placement of the bowel in a nonrotational position [2,10]. Prophylactic appendectomy is also routinely performed [9,13].

In adults who are diagnosed incidentally and are entirely asymptomatic, the role of prophylactic Ladd’s procedure remains challenging and controversial [3,6]. If obstructive symptoms are absent, corrective surgery may not be performed [9]. However, the decision should still be individualized, taking into account the patient’s overall surgical fitness and comorbidities, given that acute volvulus, even if rare, can carry significant morbidity and mortality [3,10]. In selected complex cases, additional procedures such as gastrojejunostomy and Braun anastomosis may be required [13].

The primary teaching point of this case is the importance of recognizing intestinal malrotation as an incidental finding during trauma imaging, even in the absence of gastrointestinal symptoms. In adults, the characteristic findings—such as the inversion of the SMA-SMV relationship, an abnormal duodenal course, and the malposition of the cecum—must be carefully evaluated on CT to avoid misinterpretation and to prepare for potential surgical needs in the future.

### Literature review

Multiple studies emphasize the rarity and atypical presentation of adult intestinal malrotation. Although this congenital disorder is reported in 0.1%–0.2% of the population, only a very small proportion develop clinical symptoms, which complicates diagnosis [1,3,4,6]. In one report, a 46-year-old man presented with postprandial abdominal pain, nausea, and vomiting. At laparotomy, right-sided small bowel, a centrally located cecum, and an abnormal duodenojejunal junction were observed without volvulus [6].

In another report, an 18-year-old woman with 3 years of abdominal pain and intermittent gastrointestinal symptoms had CT findings of right-sided small bowel, a left-sided cecum, and inversion of the SMA and SMV relationship, and she underwent surgical treatment [4]. In addition, a 31-year-old man evaluated for back pain and mild nausea had CT findings demonstrating the whirlpool sign and abnormal bowel positioning, and he underwent Ladd’s procedure due to volvulus [3].

Evidence indicates that contrast-enhanced abdominal CT is the preferred diagnostic tool in adults because it can accurately depict the SMA–SMV relationship as well as the whirlpool sign [2,5,[7], [8], [9]]. Given the potential for serious complications such as bowel ischemia and gangrene from volvulus, early surgical intervention—particularly using the Ladd’s procedure, which includes detorsion, division of Ladd’s bands, and mesenteric reconstruction—is critical [1,10]. In cases with borderline bowel viability, second-look surgery plays a key role in preserving bowel length and avoiding unnecessary extensive resections [1,5]. Therefore, despite its rarity, clinical suspicion for adult midgut malrotation should be maintained in adults with chronic abdominal symptoms to prevent morbidity related to delayed diagnosis and treatment [9,10].

## Conclusion

Adult midgut malrotation is a rare and likely underdiagnosed anomaly that may remain asymptomatic for years and be discovered incidentally on imaging or during surgery performed for unrelated reasons. The present case illustrates that trauma CT, even in the absence of gastrointestinal symptoms, may reveal characteristic patterns such as right-sided small bowel, left-sided cecum, an abnormal duodenal course, and an altered relationship of mesenteric vessels (ie, inversion of the normal SMA–SMV relationship, the most reliable vascular sign of malrotation). Recognition of these findings is essential to distinguish a stable, uncomplicated anomaly from acute conditions such as volvulus, obstruction, and midgut volvulus, which requires urgent intervention. Because the CT study was performed without intravenous contrast, bowel ischemia could not be definitively excluded on imaging; nevertheless, the patient’s clinical stability and absence of gastrointestinal symptoms supported an incidental, uncomplicated presentation.

Although the Ladd’s procedure is the accepted treatment for symptomatic patients or those with acute complications, the need for prophylactic surgery in completely asymptomatic adults remains debated. Accordingly, decision-making in these patients should be individualized and based on symptoms, imaging findings, the potential risks of intervention, and surgical team assessment. Patient education regarding warning signs and provision of appropriate follow-up are also key components of conservative management.

## Ethics statement

This case report was conducted in accordance with the ethical standards of the institutional and national research committee and with the 1964 Helsinki Declaration and its later amendments or comparable ethical standards.

## Declaration of generative AI and AI-assisted technologies in the writing process

An AI-based tool (GapGPT) was used for English language editing and grammar refinement. All content was reviewed and verified by the authors, who take full responsibility for the final manuscript.

## Patient consent

Written informed consent was obtained from the patient for publication of this case report and any accompanying images. A copy of the written consent is available for review by the Editor-in-Chief of this journal.
